# Rapid Prototyping of Compartmentalized 3D Microfluidic Devices for Organotypic Cell Culture

**DOI:** 10.3390/mi17050609

**Published:** 2026-05-15

**Authors:** Qasem Ramadan, Rana Hazaymeh, Mohamed Zourob

**Affiliations:** 1 College of Science & General Studies, Alfaisal University, Riyadh 11533, Saudi Arabia; mzourob@alfaisal.edu; 2 College of Pharmacy, Almaarefa University, Riyadh 13713, Saudi Arabia; rhazaimeh@um.edu.sa

**Keywords:** rapid prototyping, microfluidics, cell culture, organ-on-a-chip, 3D printing

## Abstract

We present a modular microfluidic platform for constructing miniaturized, compartmentalized cell culture systems that support monoculture, co-culture, and organ-on-a-chip models of human tissues. The devices provide architecturally defined three-dimensional microenvironments in which heterogeneous cell populations can be cultured in close proximity while maintaining precise spatial organization and independent access to each compartment. In vivo-like perfusion into, from, and between adjacent chambers is achieved via micro-engineered porous barriers that act as perfusion microchannels, enabling controlled convective and diffusive transport and recapitulating paracrine signaling between tissue units. As a proof of concept, we implement an adipose–immune co-culture model that reproduces key features of inflamed, insulin-resistant adipose tissue, including altered cytokine secretion and glucose uptake. Together, these features establish a versatile platform for the biofabrication of customizable single-organ and multi-organ in vitro models that more faithfully recapitulate human tissue structure and function for applications in disease modeling, immunometabolic studies, and preclinical drug testing.

## 1. Introduction

Organ-on-a-chip (OOC) technology provides an innovative paradigm for in vitro tissue modeling, enabling precise spatial control of cells and the establishment of in vivo-like cell polarization [1]. By offering a defined microarchitectural template, OOC platforms support the assembly of cells into complex arrangements that recapitulate the organization of native tissues. To construct organotypic cellular structures capable of physiologically relevant fluidic, biochemical, and chemical exchange, microfluidic systems are engineered to position multiple cell types in configurations that closely reflect in vivo tissue architecture. Microfluidic-based culture thereby facilitates the formation of multicellular organotypic structures, including OOC platforms and 3D constructs such as spheroids.

Although spheroids represent one important class of 3D models, native tissue architectures in vivo are rarely spheroidal [2]. Consequently, culture strategies must be optimized to promote the formation of more physiologically relevant cell assemblies and their associated extracellular matrix, which are critical for achieving in vivo-like function prior to downstream analysis. Advances in microfluidic technology now permit the integration of multiple tissue types or organ models within a single perfused circuit, enabling controlled organ–organ crosstalk while preserving the discrete functionality of each unit and emulating vascular perfusion. OOC systems thus support dynamic interactions between diverse cell populations and allow time-resolved measurements at defined checkpoints, providing mechanistic insight into complex and evolving biological processes [3,4].

The successful design of a compartmentalized fluidic system for OOC technology is crucial for establishing a physiologically relevant and effective in vitro model. This design requires careful consideration of several critical parameters, including the appropriate size of individual compartments, which must correspond to the size of the hosted organ. Furthermore, the order in which the organs are connected, the tissue orientation, and the perfusion rate within each compartment require careful evaluation.

To establish an organotypic structure and facilitate functional tissue coupling that supports cell nutrient supply, chemical signaling, and paracrine communication, OOC systems employ various connection strategies: (i) Convection-based fluidic transfer, which can be achieved through manual pipetting or the use of tubing. This connection method does not rely on microfabricated channels to link the fluidic chambers [5,6,7,8,9,10,11,12]. However, this approach has notable limitations, primarily its inability to fully recapitulate the physiological flow between organs. Additionally, this method is restricted to the use of organs that can be maintained with the same culture medium. The arrangement of cell types in this design does not accurately replicate the in vivo organization, where there is often a significant distance between different cell types in terms of cell–cell interfacing (e.g., through channels or tubing). While this design is well-suited for connecting cells or tissues separated by a certain distance, such as heart–liver or intestine–liver connections, it fails to provide a physiologically relevant cell–cell interface for modeling the complex cellular structure of a single organ, such as the liver, which comprises multiple cell types (e.g., hepatocytes, hepatic stellate cells, sinusoidal endothelial cells, and Kupffer cells). Furthermore, it does not support interactions between parenchymal and non-parenchymal cells or immune cells, where close proximity between cells is essential. A few commercial platforms have become available to address these limitations [13]. (ii) Utilizing vertical porous barriers or gel. In this configuration, cells are assembled and cultured in a two-dimensional (2D) arrangement within two or three planar compartments separated by semi-porous vertical barriers [14,15,16,17]. This arrangement allows different cell types to be cultured in close proximity, ensuring physical isolation while maintaining fluidic and chemical connectivity between compartments. The horizontal order of cell co-culture is a commonly employed structure in OOC systems. This arrangement is preferred due to its ease of fabrication and suitability for studying cell–cell interactions. Although this horizontal configuration supports close-proximity co-culture and is favored for its relative fabrication simplicity, it is inherently constrained to simple cell culture organization, mainly two-cell type culture, and offers limited control over the directionality and magnitude of inter-compartmental transport. (iii) Using porous membranes. In this configuration, two fluidic compartments are vertically stacked and connected via a porous membrane. This organization allows for the co-culture of two or more cell types in close proximity within a vertical orientation. By culturing epithelial, endothelial, or epidermal cells on the upper surface of the membrane and the corresponding parenchymal tissue on the lower side, this structure closely mimics the architecture and functionality of critical biological barriers in the human body. These barriers include the small intestine, lung parenchyma, skin, and blood vessels, which play essential roles in regulating interactions between the body and external factors such as drugs, food, and environmental agents [18,19,20,21,22,23,24]. This configuration is architecturally restricted to bilayer systems and does not readily support the simultaneous co-culture of three or more spatially distinct cell populations within a single chip, nor does it allow the flexible customization of compartment geometry that is required to model the complex multicellular microenvironment of individual organs such as the liver or adipose tissue.

Several compartmentalized microfluidic platforms employing porous barriers have been reported for organotypic co-culture. Early planar compartmentalized systems, such as those of [25] and Liu et al. [14], demonstrated semi-porous sidewall barriers for co-culture models but were fabricated using conventional soft lithography or laser-micromachined PMMA and constrained to simple two- or three-compartment linear geometries with fixed, uniform pore distributions. In the domain of 3D-printed microfluidics, while DLP, SLA, and FDM approaches have been explored for OOC devices [26], and isolated studies have incorporated rudimentary porous features into printed chips [27], none have combined within a single monolithic architecture the ability to freely vary compartment topology, selectively position pores at defined wall heights, and support perfusion-controlled crosstalk among three or more spatially distinct cell populations. Commercial platforms including MIMETAS OrganoPlate^®^ [28,29,30,31,32,33], TissUse HUMIMIC [34,35,36], and Emulate organ chips [37,38,39,40] each represent important advances but remain restricted to fixed geometries—gel/perfusion lane formats, endothelial-lined channel connections, or bilayer membrane architectures—that preclude freely configurable planar multi-tissue arrangements.

We have designed and fabricated a compartmentalized perfusion device that serves as a cell culture template, enabling the co-culturing of multiple cell types in the desired spatial organization or physiologically relevant architectures. This improves the cell–cell and tissue–tissue interactions, allowing the recapitulation of the structure of an individual human organ or a network of organs, so that metabolites and paracrine signals can be transported and exchanged between various tissues or organ models. Unlike conventional porous membrane or gel-barrier systems, the PB architecture introduced in this work offers a substantially expanded design space. Barrier walls can be oriented perpendicular, parallel, curved, or in meandering configurations relative to the main flow axis, and pores can be selectively positioned at the top, bottom, mid-height, or distributed across the full wall height, affording precise spatial control over inter-compartmental transport across both diffusive and convective regimes. This geometric flexibility enables a range of multi-compartment topologies, including linear, interdigitated, and honeycomb arrangements, to be realized within a single planar chip without cleanroom processing, photolithography, or multi-layer bonding. The entire PB design space, encompassing barrier orientation, pore placement, wall topology, and compartment order, is accessible through a single DLP 3D-printing workflow, making the platform rapidly reconfigurable to suit diverse organotypic co-culture requirements.

It is important to note that the primary focus of this work is on the engineering aspects, specifically the design and fabrication of the microfluidic chip. The scope of the study is primarily limited to these areas. However, basic system characterization is also conducted, including relevant cell co-culture experiments. While the emphasis is on the engineering aspects, the paper provides a preliminary understanding of the system’s performance in a biological context. It serves as a foundation for further research and exploration in the field of cell co-culture using microfluidic systems.

## 2. Materials and Methods

### 2.1. Materials

The poly(methyl methacrylate) (PMMA) sheets with thicknesses of 0.5 mm, 1 mm and 2 mm were purchased from a local vendor. Double-sided adhesive (3M 467MP Adhesive Transfer Tape Acrylic 2.3 mil) was from 3M (Saint Paul, MN, USA). 3D printing resin (DentaGuide) from Asiga (Alexandria, NSW, Australia). Human pre-adipocytes (HPAd) cat # 802s-05a, pre-adipocyte growth medium, adipocyte differentiation medium, adipocyte maintenance medium, and adipocyte starvation medium from Cell Applications (San Diego, CA, USA); iMDM medium from Thermo Fisher Scientific (Dubai, United Arab Emirates); Lipid A (LPA) from E.coli serotype R515 (TLR grade) from Alexis Biochemials (San Diego, CA, USA); TNF-α, IL-6 and IL-8 ELISA kits and Calceine-AM from ThermoFisher Scientific (Dubai, United Arab Emirates); 2-NBDG glucose uptake cell-based assay kit from Cayman Chemical (Ann Arbor, MI, USA); Hoechst 33342 from Thermo Fisher Scientific (Dubai, United Arab Emirates).

### 2.2. Design of the Systems

In this study, we evaluate the utility of the 3D printing technique to fabricate complex compartmentalized microfluidic structures with various compartment arrangements and functionalities for cell organotypic culture. A key component of these compartmentalized devices is the porous barriers (PBs), which act as a physical barrier to retain cells of a certain type within a specific compartment while enabling fluid exchange between the adjacent compartments in various connection modes. The multi-compartment microfluidic systems are designed such that each compartment hosts a distinct organ/tissue model. Due to the miniaturized nature of the microfluidic system and by maintaining a continuous flow of culture media into the cell culture chamber, the ratio between the cell volume and media can be reduced to a minimum to simulate the in vivo environment. For example, when cells are cultured in a conventional culture flask with a surface area of 75 cm^2^, 10 mL of culture media is added, resulting in a media/cell ratio (volume/volume) of 67. In contrast, when the same cells are cultured in a microfluidic chip with a surface area of 10^−2^ cm^2^, the volume of media covering the cells is within the range of 20–30 μL, resulting in a ratio of media/cell of only 6. Furthermore, the cell culture compartments that represent the multi-organs can be organized in a hierarchy that mimics the physiological order, which may allow tracking the key physiological events in disease progression and treatment.

**Figure 1:**
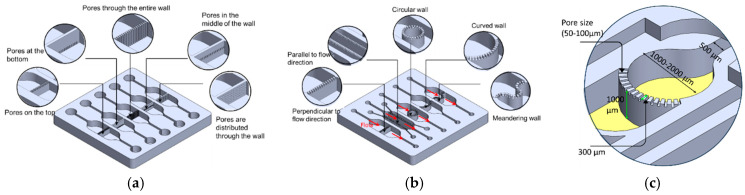
(**a**) PBs are characterized by an array of small pores that are located either (1) in the upper or (2) the lower side of the walls (3) through the entire wall height, (4) in the middle of the barrier wall or (5) distributed through the entire surface of the wall. (**b**) The PBs can be fabricated in various orientations such as perpendicular to the flow direction, parallel to the flow direction, a single curved wall, and a circular and a meandering wall. (**c**) The dimensions of the critical components.

PBs consist of arrays of small pores that can be positioned on the upper or lower surface of the wall, span the full wall height, be confined to the mid-height region, or be distributed across the entire wall, as illustrated in Figure 1a. The pore arrangement can be tailored to specific experimental requirements so that cells are retained within their designated compartments while permitting controlled chemical and biological interactions (paracrine signaling) between neighboring compartments. Each pore effectively forms a short perfusion micro-channel that hydraulically connects two adjacent compartments. The rate and direction of this inter-compartmental flow are regulated by external pumps, allowing heterotypic cell–cell communication to be modulated via micro-scale perfusion through the PBs.

**Figure 2:**
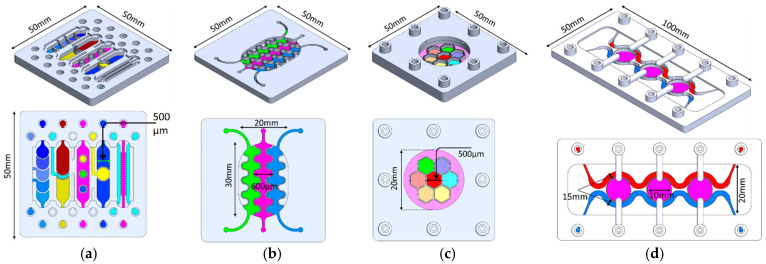
(**a**) Linear order: The compartments are arranged in series in the same direction of the flow. The PBs can be straight, curved or circular. (**b**) Interlaced arrangement along the flow direction. (**c**) Honeycomb structure: A central hexagonal compartment surrounded by 6 hexagonal compartments. (**d**) Tri-compartment device comprises three central chambers interfaced with two perfusion channels. All the compartments are fluidically connected through the porous walls.

PBs can be implemented in multiple orientations relative to the main flow, including: (1) perpendicular to the flow direction, (2) parallel to the flow direction, (3) single curved walls, (4) meandering walls, and (5) circular configurations (Figure 1b). This versatility in PB geometry and placement supports the design of highly customized, compartmentalized microfluidic architectures for studying multicellular interactions and tissue-level phenomena. To demonstrate the manufacturability of these designs, PBs were implemented in several exemplary organizational layouts:

(1) Linear arrangement: Compartments are aligned in series along the flow direction, with PBs that can be straight, curved, or meandering (Figure 2a).
(2) Interdigitated arrangement: Meandering PBs generate interdigitated compartments, enhancing biochemical signal exchange between adjacent regions (Figure 2b).
(3) Honeycomb arrangement: A central hexagonal compartment surrounded by six neighboring hexagonal compartments, all fluidically interconnected via PBs (Figure 2c).
(4) Tri-compartment perfusion device: This device comprises three independent circular compartments interfaced with two side channels through the PBs (Figure 2d).

### 2.3. Fabrication

**Figure 3:**
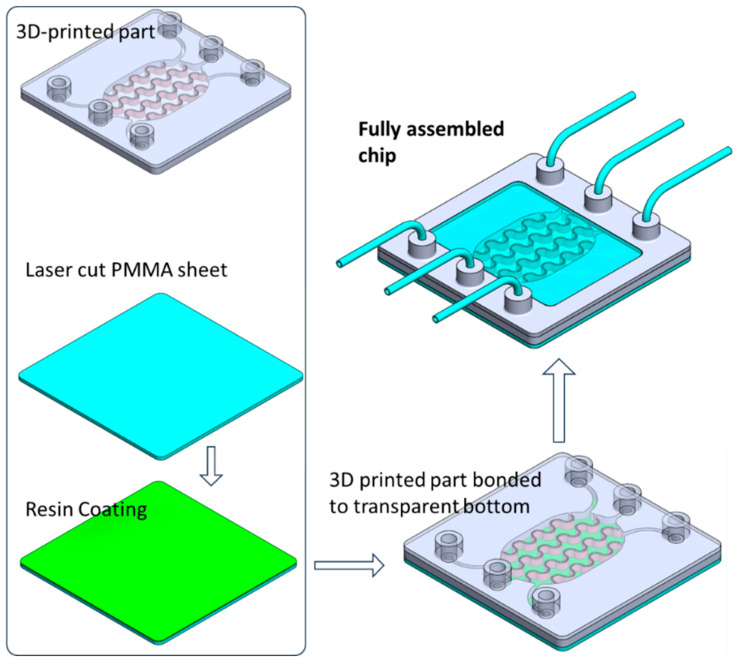
Schematic illustration of the fabrication and assembly of the organ-on-a-chip device, showing the 3D-printed microfluidic component, laser-cut PMMA substrate with resin coating, bonding of the printed part to the transparent bottom layer, and the final assembled chip with integrated perfusion inlets and outlets.

The device was designed using SolidWorks, version 2021 (Dassault Systèmes, Vélizy-Villacoublay, France) and fabricated with a digital light processing (DLP) 3D printer (ASIGA MAX UV, Alexandria, NSW, Australia). DLP printing employs a projected light source to photopolymerize a liquid resin layer by layer, thereby generating the three-dimensional structure. The CAD model was exported as an STL file, processed with the slicer software (Asiga Composer from ASIGA, Alexandria, NSW, Australia) to produce a stack of PNG layer images, and subsequently uploaded to the printer. A biocompatible photopolymer resin (DentaGuide, from ASIGA, Alexandria, NSW, Australia) was loaded into the printing tank, and the build was initiated. To enable high-quality imaging of cells within the chip, the bottom layer was required to be optically clear and transparent. This layer was therefore fabricated from PMMA using computer-assisted CO_2_ laser cutting (Beambox, Flux, Taipei, Taiwan), following conversion of the design to a DXF file. After 3D printing of the main structure and laser cutting of the PMMA base, the two components were bonded using the same 3D printing resin as an adhesive. A thin resin film was first deposited onto a PMMA sheet via spin coating, after which the 3D-printed part was gently pressed onto the coated surface to transfer a uniform resin layer. The assembled chip was then exposed to UV light for 1 h to cure the resin and achieve permanent bonding. The overall fabrication workflow is schematically illustrated in Figure 3.

### 2.4. Characterization of the Flow Within the Devices

The fluidic ports of the device were connected to a set of syringes, which were mounted on a programmable syringe pump (KD Scientific, Holliston, MA, USA) through PEEK tubing with an inner diameter of 0.5 mm. To visualize the flow profile within the device, colored liquid was injected into the inlet of a selected chamber, and the infusion of the liquid into the subsequent chambers was monitored over time. A series of images was taken to track the flow profile across the multi-compartment devices. To examine the mass transfer between adjacent chambers, the transport of FITC–dextran (4 KDa) tracer through the porous barriers was monitored by injecting the tracer solution with a concentration of 10 µL/mL in a selected upstream chamber, which had been initially filled with phosphate-buffered saline (PBS) solution. The fluorescence intensity due to the diffusion of the tracer was measured at different downstream checkpoints using a fluorescent plate reader (PerkinElmer, Richmond, CA, USA).

### 2.5. Cell Culture: In Vitro Model of the Inflamed Human Adipose Tissue

The interdigitated chip was selected to co-culture human adipocytes and human monocytic cells. This in vitro model recapitulates some features of the complex cellular environment found in inflamed adipose tissue, which enables studying the mechanisms underlying insulin resistance.

Prior to seeding the cells into the chip, the fluidic compartments underwent a sterilization process by filling them with 70% ethanol for a minimum of 4 h. Afterward, the chips were washed with deionized water, and each chip was loaded with 100 µL of Poly-L-lysine solution (0.1 mg/mL in H_2_O) and incubated overnight at 37 °C. The chips were then washed again, first with sterilized deionized water and then with phosphate-buffered saline (PBS). Next, the chips were primed with a preadipocyte growth medium (Cell Applications, San Diego, CA, USA). A suspension of Human Preadipocytes (HpA) with a cell density of 1.5 × 10^6^ cells/mL and cell viability above 85% was inoculated into the side compartments. The cells were allowed to attach under static culture conditions without perfusion for 2 h in a 5% CO_2_ humidified atmosphere. Following cell attachment, perfusion was initiated at a flow rate of 8 nL/s. The preadipocytes were cultured under perfusion conditions until they reached confluence, which typically took around 2–3 days. To induce cell differentiation, the preadipocyte growth medium was replaced with an adipocyte differentiation medium (#811D-250, Cell Applications). After 12–14 days of differentiation, an adipocyte maintenance medium (#811M-250) was applied for at least two days before further cell characterization or co-culture with immune cells. Morphological images of the cells were acquired throughout the process. Lipid droplets were observed in the cells after approximately 2 days of differentiation. To assess cell differentiation of the adipocytes, the lipid content within adipocytes was monitored by staining the lipid droplets with Oil Red O (Sigma-Aldrich, St. Louis, MO, USA). The cells were initially fixed with 4% paraformaldehyde (PFA) and subsequently treated with the Oil Red O solution for a duration of 30 min at room temperature. Afterward, the cells were washed with deionized (DI) water. Bright-field images were captured using a microscope.

The human leukemic monocyte lymphoma cell line (U937) (Addexbio Biotechnologies, San Diego, CA, USA) was used as a model of human immune responsive dendritic cells (DCs). Once the adipocytes were fully differentiated, U937 cells were collected and suspended in an adipocyte maintenance medium. A 100 µL suspension of U937 cells was then inoculated into the central compartment of the chip. The adipocyte maintenance medium was continuously perfused through the three compartments at a flow rate of 8 nL/s for 2–3 days.

This flow rate was selected based on two criteria: (i) it produces a calculated wall shear stress of approximately 0.01–0.05 dyn/cm^2^ within the cell culture compartments (consistent with values used in adipose tissue microfluidic models); and (ii) it maintains a sufficient media turnover rate to prevent nutrient depletion over the 3-week culture period, as estimated from the compartment volume and metabolic consumption rates reported for adipocytes in the literature.

Cell viability and morphological changes were monitored using a microscope. A mixture of calcein-AM (2 µM) and ethidium homodimer-1 (4 µM) diluted in Dulbecco-PBS (D-PBS) was used to assess cell viability. Then the cell viability was examined using a fluorescent microscope. Cell viability was calculated as the percentage of calcein AM-labeled cells. Images were captured from various locations within the cell compartment and the percentage values obtained from these images were then averaged. Data were represented as mean ± SD.

To induce an inflammatory state, the cells were treated with lysophosphatidic acid (LPA) at a concentration of 100 ng/mL. Supernatants from the sampling outlet were collected for further analysis. The concentrations of Tumor Necrosis Factor (TNFα) and Interleukin-6 (IL-6) in the supernatants were measured using an enzyme-linked immunosorbent assay (ELISA) method. To serve as controls, experiments were also conducted on untreated samples and adipocyte monocultures.

### 2.6. Biochemical Characterization

Glucose uptake by the adipocytes: The glucose uptake by the differentiated adipocytes was measured using a glucose uptake assay kit. This kit employs 2-deoxy-2-[(7-nitro-2,1,3-benzoxadiazol-4-yl)amino]-D-glucose (2-NBDG) as a fluorescent-labeled deoxyglucose analog probe (Cayman Chemicals, Ann Arbor, MI, USA).

Cytokine immunoassay: The detection of inflammatory cytokines released from the co-cultured cells was performed using ELISA. After the treatment, the supernatant from the chip was collected at different time intervals for analysis. The expression of the inflammatory cytokines, TNF-α and IL-6, was investigated. Approximately 100 μL of the supernatant was collected from each chip/experiment for subsequent ELISA testing. The cytokine concentrations in the medium were measured using the ELISA, following the manufacturer’s instructions. Briefly, antibody-coated 96-well microplates were first coated with a primary antibody. The supernatant solution was then injected into the antibody-coated microplates and incubated. The plate was washed, and a biotin-labeled detection antibody was added, followed by streptavidin-HRP. Finally, the absorbance was measured using a plate reader (Perkin Elmer, Waltham, MA, USA).

## 3. Results

### 3.1. Fluid Flow Visualization

**Figure 4:**
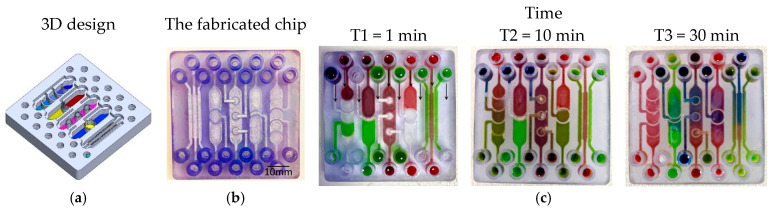
Test structures of various designs of PBs between multi-compartments. (**a**) The 3D structure design. (**b**) The fabricated chip. (**c**) A series of images showing the color diffusion and mixing through the PBs between different compartments.

**Figure 5:**
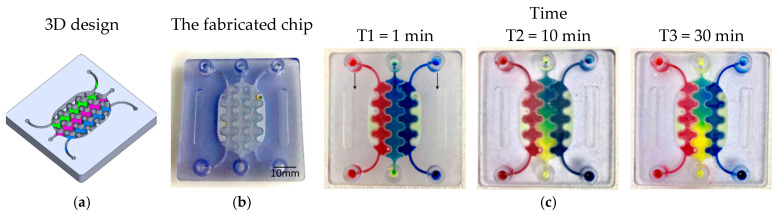
A compartmentalized microfluidic chip with 3 planar compartments separated interlaced PBs. (**a**) 3D view of the designed system.(**b**) The fabricated chip. (**c**) A series of images showing the color diffusion through the PBs between different compartments.

**Figure 6:**
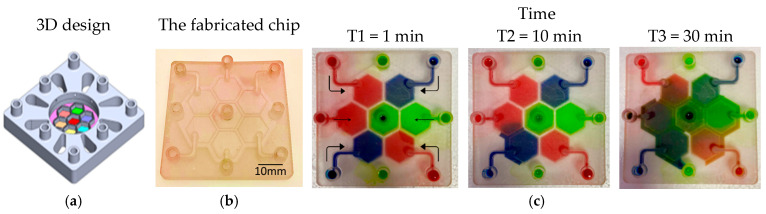
Five interconnected compartments arranged in a honeycomb-shaped structure. The compartments are separated by PBs. (**a**) The 3D structure design. (**b**) The fabricated chip. (**c**) A series of images showing the color diffusion through the PBs between different compartments. Arrows indicate the flow direction in the channels.

**Figure 7:**
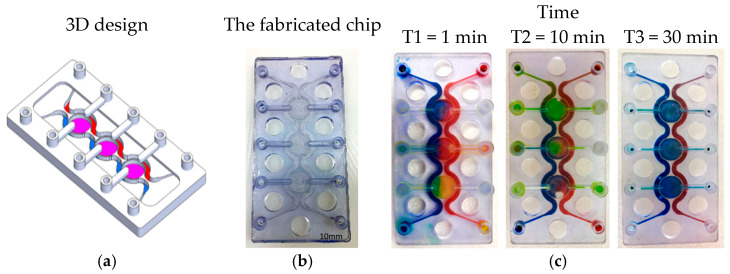
Tri-compartment perfusion device. (**a**) The designed chip. (**b**) The fabricated chip. (**c**) A series of images showing the color diffusion through the SPWs between different compartments.

To visualize and qualitatively validate fluid transport within the fabricated chips, colored aqueous solutions (water containing food dyes) were introduced into individual inlet ports, and the subsequent redistribution of dye between compartments was monitored over time. Sequential images acquired at defined time points (Figure 4, Figure 5, Figure 6 and Figure 7) capture the evolution of the color fronts as they propagate across the porous barriers, providing a direct readout of inter-compartmental diffusion and convective exchange. In all three designs—linear, interdigitated, and honeycomb—the progressive mixing of differently colored streams confirms successful fluidic connectivity between neighboring compartments and illustrates how the device geometry modulates the kinetics and spatial pattern of solute exchange.

### 3.2. Inter-Compartment Permeability

**Figure 8:**
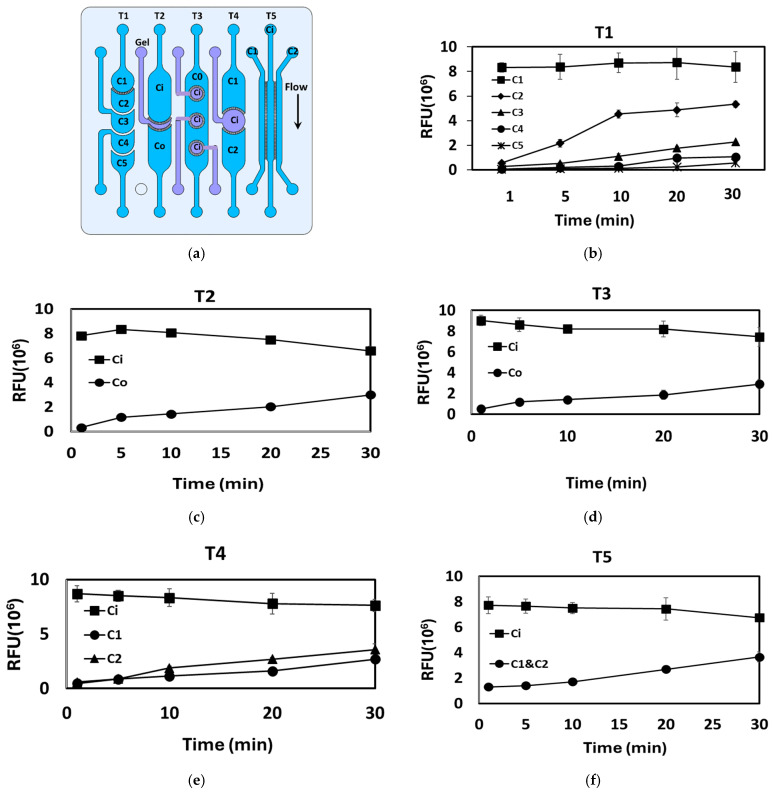
(**a**) Drawing of the five different designs (T1 to T5) illustrating the location of the donor and acceptor compartments and the route of the tracer transport. (**b**–**f**) The relative fluorescence intensity measured in the various downstream compartments after injection of 4 KDa FITC–dextran at a concentration of 5 μg/mL in the inlet compartment. Ci: Inlet chamber; Co: Outlet chamber.

The crosstalk between various adjacent compartments in the fabricated devices was visualized by injecting FITC–dextran 4 kDa tracer into one compartment (upstream) at a concentration of 5 µg/mL and recording the FI across the downstream compartments at different time intervals. Figure 7 and Figure 8 show the permeability profile between the various adjacent compartments as indicated by the FIs. The inset on the right depicts the route of interest for FITC transport. Prior to injection of the FITC–dextran solution, the chips were initially filled with a clear PBS solution. Figure 8 shows a series of linear fluidic structures, which consist of various fluidic compartments arranged in a linear configuration and separated by PBs. The five designs are labeled as T1, T2, T3, T4, and T5 (Figure 8a). To evaluate the compartmental connectivity, a FITC–dextran solution was injected into the upstream inlets, and the FIs were measured at the different downstream compartments. The results are presented in Figure 8b–f and summarized in Table 1. PBs with different pore distribution as shown in Figure 1a were tested. The location/distribution of the pores within the PBs does not appear to have a substantial impact on the compartmental connectivity and the ability of the tracer or color to permeate across the barriers.

**Figure 9:**
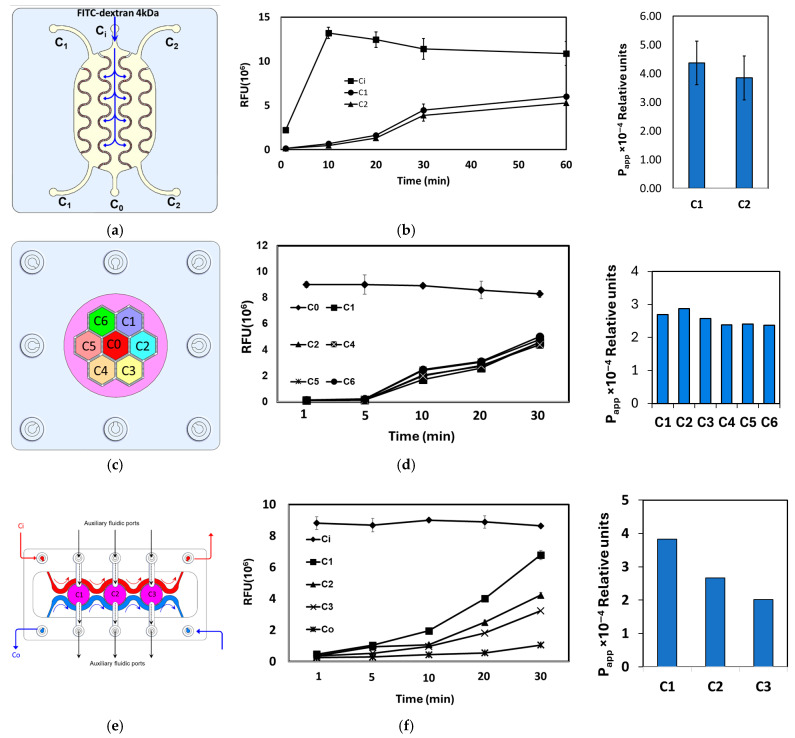
(**a**,**c**,**e**) Top view drawing of the interdigitated, honeycomb and tri-compartment perfusion chip designs illustrating the location of the donor and acceptor compartments and the route of the tracer transport. (**b**,**d**,**f**) The relative fluorescence intensity measured in the various downstream compartments after injection of 4 KDa FITC–dextran at a concentration of 5 μg/mL in the inlet compartment.

In the interdigitated design (Figure 9a,b), the FITC–dextran tracer was introduced into the central donor compartment (C0), and fluorescence intensity (FI) was monitored in both the donor and the adjacent acceptor compartments (C1 and C2). The low initial FI in C1 and C2, followed by a steady, time-dependent increase, confirms efficient inter-compartmental mass transport and chemical crosstalk across the porous barriers.

In the honeycomb design (Figure 9c,d), the tracer was similarly injected into the central donor compartment (C0) and FI was quantified in the six surrounding acceptor compartments (C1–C6). As in the interdigitated chip, FI was initially high in C0 and close to the background in C1–C6, then progressively increased in all peripheral compartments, demonstrating robust radial diffusion and convective exchange throughout the honeycomb network.

The tri-compartment perfusion device (Figure 9e,f) comprises three central circular chambers (C1–C3) separated by porous barrier walls and flanked by two longitudinal perfusion channels: an inlet channel (Ci) delivering tracer solution and an outlet channel (Co) for waste removal and sample collection. Each chamber is additionally accessible via its own auxiliary inlet and outlet port, enabling independent seeding or stimulation. Upon injection of FITC–dextran into Ci, the tracer gradually permeated through the porous barriers into chambers C1–C3 and, with further perfusion, into the outlet channel Co, as reflected by the corresponding FI–time profiles. Together, these measurements demonstrate controlled, time-resolved crosstalk between adjacent compartments in all three microfluidic architectures.

### 3.3. Cell (Co)-Culture Characterization

Cell culture and co-culture experiments were implemented in two selected devices—the interdigitated and the tri-compartment perfused devices. This was done to examine the crosstalk between the grown cells, with the aim of modeling a specific (patho)-physiological condition, which is insulin-resistant in inflamed adipose tissue.

**Figure 10:**
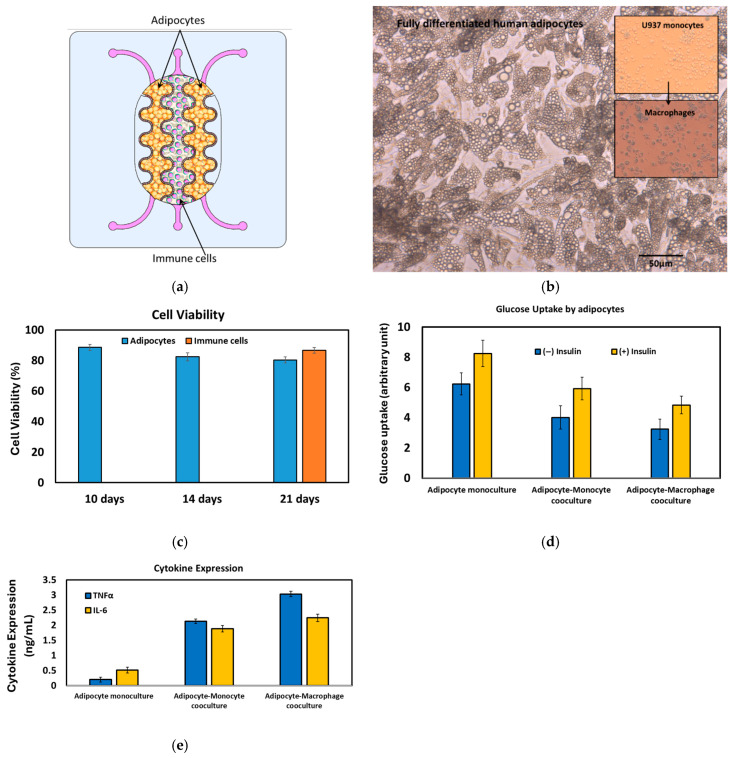
(**a**) Schematic drawing of the adipocyte-immune cell co-culture for modeling the immune cell (macrophage) infiltration in the insulin resistance. (**b**) Fully differentiated and hypertrophied adipocytes after 20 days of culture. U937 monocytes and U937-based macrophages are shown in the inset. (**c**) Cell viability of the co-cultured cells. (**d**) Glucose uptake by the adipocytes at three different cell culture setups. (**e**) Cytokine expression at three different cell culture setups.

*The interdigitated device*: Infiltration of immune cells, particularly monocytes, macrophages, and Th1 cells, into adipose tissue is associated with chronic low-grade inflammation in obese individuals [41,42]. These inflammatory immune cells interact with adipocytes, triggering chronic inflammation that ultimately leads to the impairment of insulin action and the development of insulin resistance [43,44]. Macrophages are the most abundant immune cell type present in obese adipose tissue, comprising up to 50% of the total cell population [45], compared to only around 5% in lean adipose tissue. The expansion of adipose tissue in obesity disrupts the normal secretion of adipokines like MCP-1 and adiponectin, further inducing monocyte infiltration into the adipose tissue [46,47]. To demonstrate the practical application of the fabricated devices, the interdigitated structure was used to create an organotypic co-culture system. In this system, human pre-adipocytes were cultivated in the outer compartments (C1 and C2), while human monocytes (U937 cell line) were seeded into the central compartment (C0) (Figure 10a). This co-culture model aims to mimic the relevant microenvironment found in inflamed human adipose tissue, allowing for the study of the crosstalk between adipocytes and infiltrating immune cells.

The co-culture of adipocytes and immune cells was maintained for three weeks prior to the immune-metabolic analysis. Fully differentiated adipocytes were consistently observed after 14–20 days of culture (Figure 10b). Cell viability remained above 80% throughout the 21-day culture period (Figure 10c). To induce an inflammatory state, monocytic cells were treated with 100 ng/mL of lysophosphatidic acid (LPA). From the same set of adipocytes, both glucose uptake and the secretion of TNFα and IL-6 into the supernatants were quantified. The immune-metabolic status of the co-culture was evaluated (Figure 10d,e). A correlation was observed between the cytokine profile and glucose uptake. In the adipocyte monoculture, significant glucose uptake occurred, particularly following insulin treatment, with negligible cytokine regulation. Conversely, in the co-culture system, a slight reduction in glucose uptake was accompanied by a notable increase in cytokine secretion. Furthermore, when the inflammatory state was induced by LPA treatment, promoting differentiation into macrophages, there was a marked increase in cytokine secretion and a significant reduction in glucose uptake. The interaction between human adipocytes, immune cells, and tissue-resident macrophages provides a robust model for investigating how the interplay among different cell types contributes to the pathogenesis of various diseases, including type 2 diabetes.

**Figure 11:**
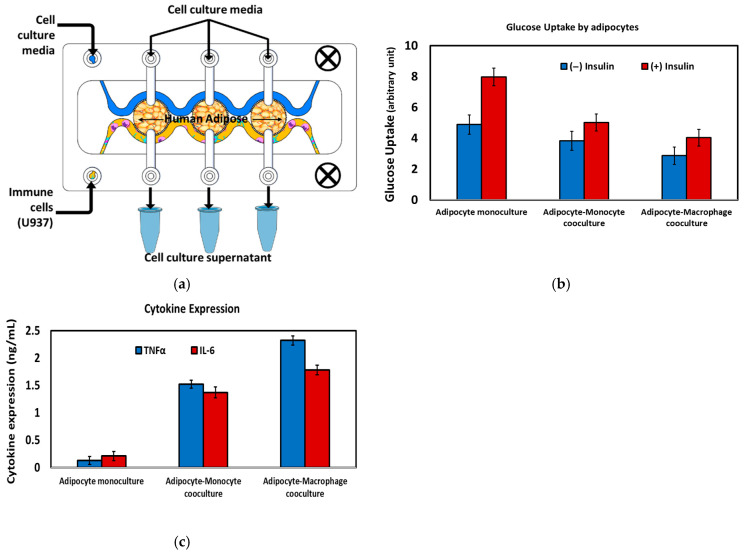
(**a**) Schematic drawing of the adipocyte-immune cell co-culture for modeling the immune cell (macrophage) infiltration in the insulin resistance. (**b**) Glucose uptake by the adipocytes at three different cell culture setups. (**c**) Cytokine expression at three different cell culture setups.

*The tri-compartment device*: In the tri-compartment perfusion device, human adipocytes were first cultured and differentiated within the three central chambers (C1, C2, and C3) under continuous perfusion (Figure 11a). After establishing a stable adipose tissue layer, monocytic U937 cells were introduced via the outlet channel (Co) and differentiated on-chip into macrophages via lipopolysaccharide (LPA) stimulation, thereby modeling immune-cell infiltration into adipose tissue. A key feature of this microfluidic configuration is the ability to maintain adipocytes and immune cells in spatially separated but fluidically connected compartments, enabling sustained exchange of soluble mediators while preventing direct cell overgrowth or displacement. This arrangement recreates an in vivo-like inflammatory adipose microenvironment and permits time-resolved sampling of the cell culture supernatant from each chamber for downstream biochemical analysis (Figure 11a).

The immunometabolic status of the cultures was quantified by measuring insulin-stimulated glucose uptake in adipocytes and secreted cytokines (TNF-α and IL-6) under three conditions: adipocyte monoculture, adipocyte–monocyte co-culture, and adipocyte–macrophage co-culture (Figure 11b,c). In adipocyte monoculture, cells exhibited robust insulin-responsive glucose uptake with minimal cytokine release, consistent with a non-inflamed, insulin-sensitive phenotype. Introduction of immune cells led to a progressive reduction in glucose uptake and a concomitant increase in pro-inflammatory cytokine secretion, most pronounced in the adipocyte–macrophage co-culture, reflecting the well-described link between macrophage-derived inflammatory mediators and adipocyte insulin resistance. These findings demonstrate that these devices support controlled adipocyte–immune cell crosstalk and recapitulates key features of inflamed, insulin-resistant adipose tissue in vitro.

## 4. Discussion

The microfluidic systems presented in this work expand the palette of available organ-on-chip architectures by introducing a family of planar, compartmentalized devices with highly tunable porous barriers and fluidic layouts. In contrast to many existing platforms that rely either on simple tubing-based interconnections or on fixed membrane inserts, our approach allows precise control over inter-compartmental distance, barrier geometry, and pore distribution within a single monolithic chip, thereby enabling systematic modulation of convective and diffusive coupling between adjacent tissue compartments. This flexibility is critical for recapitulating the broad spectrum of cell–cell interfaces encountered in vivo, ranging from tightly apposed epithelial–endothelial contacts to more distant parenchymal–immune cell interactions within complex organs such as adipose tissue or liver.

Planar compartmentalization using semi-porous sidewalls provides several advantages over conventional stacked membrane configurations. First, all compartments reside in a single optical plane, which simplifies high-resolution imaging, facilitates multi-region time-lapse microscopy, and improves compatibility with standard inverted microscopes and high-content imaging platforms. Second, the porous barriers act as short perfusion microchannels with a defined length equal to the wall thickness, allowing the hydraulic resistance and mass-transfer characteristics between compartments to be engineered by adjusting wall thickness, pore diameter, and pore density. Third, lateral co-culture in a shared plane enables straightforward integration of additional sensing modalities (e.g., impedance or TEER electrodes, optical readouts) without the alignment constraints of vertically stacked devices. Together, these features position the platform as a versatile testbed for dissecting how micro-architecture and flow govern paracrine signaling and tissue-level responses.

The versatility of the compartmentalized platform described here stems not only from its fabrication flexibility but also from the capacity of each PB geometry to be matched to a specific biological architecture and the physiological communication mode it is intended to recapitulate. The linear arrangement, in which compartments are aligned in series along the flow direction, is particularly well-suited for modeling sequential organ axes such as the gut–liver or intestine–kidney, where directional and unidirectional chemical exchange reflects the physiological reality of luminal contents or absorbed metabolites passing through successive tissue barriers before reaching downstream organs. In this configuration, soluble mediators, nutrients, and drug metabolites generated in an upstream compartment are transported convectively through the PBs into adjacent downstream compartments in a manner that mirrors the portal venous flow connecting the intestinal epithelium to the hepatic parenchyma in vivo. The interdigitated arrangement, by contrast, maximizes the shared interfacial area between adjacent compartments through its meandering barrier geometry, generating a high-surface-area contact zone that sustains dense bidirectional paracrine exchange. This configuration is ideally suited for co-cultures in which intimate, reciprocal signaling between two cell populations is the primary biological variable under investigation, as exemplified in the present study by the adipocyte–monocyte–macrophage co-culture model of inflamed adipose tissue. The pronounced increase in pro-inflammatory cytokine secretion and concomitant reduction in insulin-stimulated glucose uptake observed in the adipocyte–macrophage co-culture condition are consistent with the well-described role of macrophage-derived TNF-α and IL-6 in driving adipocyte insulin resistance [48] and reflect the biological advantage conferred by the interdigitated geometry in sustaining this crosstalk over a 21-day culture period. Finally, the honeycomb architecture, in which a central compartment is surrounded by six fluidically connected peripheral compartments, enables radially symmetric, multi-directional crosstalk among three or more distinct cell populations simultaneously. This geometry is particularly appropriate for modeling tissue microenvironments characterized by a central functional unit surrounded by supporting or interacting cell types, such as the hepatic lobule, where a central vein is flanked by radially arranged hepatocytes, stellate cells, Kupffer cells, and sinusoidal endothelial cells, or secondary lymphoid structures such as the lymph node cortex, where antigen-presenting cells, T cells, and B cells occupy spatially defined but intercommunicating niches. Together, these geometry-to-application mappings illustrate that the choice of PB architecture is not merely an engineering preference but a biologically motivated design decision that directly determines the mode, directionality, and spatial resolution of inter-compartmental crosstalk achievable within the platform.

An important contribution of this work is the demonstration that the same design rules can be used to generate a spectrum of compartment arrangements—from simple two-chamber layouts to interdigitated, honeycomb, and multi-chamber perfusion geometries—using a unified fabrication workflow based on DLP 3D printing and laser-cut thermoplastics. The interdigitated and honeycomb structures illustrate how increasing the interfacial area between donor and acceptor compartments, while maintaining physical segregation of the cell populations, enhances molecular exchange and supports more homogeneous exposure of tissues to soluble cues. The tri-compartment perfusion device, in turn, exemplifies how parallel chambers can be coupled to common inlet and outlet channels to impose defined concentration gradients or synchronized stimulation while still permitting chamber-specific seeding and readout. Because all of these designs rely on the same modular porous-barrier concept, they can be readily adapted to different organotypic models by tuning chamber volume, barrier length, and connectivity to reflect organ size, vascular topology, and physiological flow patterns.

The proof-of-concept adipose–immune co-culture experiment further underscores the biological relevance of the platform. By hosting differentiated human adipocytes in dedicated chambers and exposing them to U937-derived macrophages via controlled perfusion, the system reproduces key features of inflamed adipose tissue, including elevated pro-inflammatory cytokine secretion and impaired insulin-stimulated glucose uptake. This immunometabolic coupling is difficult to capture in conventional static co-culture formats, where concentration gradients collapse and the ratio of cell volume to medium volume is far from physiological. In our devices, the reduced media-to-cell volume ratio and continuous perfusion more closely approximate in vivo exposure conditions, thereby providing a more sensitive readout of subtle changes in adipocyte function. Such disease-relevant responses highlight the potential of these compartmentalized chips as screening tools for anti-inflammatory or insulin-sensitizing interventions.

From a translational perspective, the modularity of the compartmentalized layout is particularly advantageous for constructing multi-organ microphysiological systems. Because individual chips can be fluidically bridged in series or parallel, it becomes feasible to assemble networks that recapitulate physiologically ordered organ axes (e.g., gut–liver–adipose) while retaining the ability to characterize each unit independently. The capacity to independently tailor barrier porosity and flow between any pair of compartments offers a route to emulate organ-specific residence times, shear stresses, and metabolite distributions, which are critical determinants of drug disposition and toxicity. Furthermore, the rapid, mask-free fabrication afforded by DLP 3D printing lowers the barrier to iterative design, enabling laboratories without access to cleanroom facilities to prototype custom architectures matched to specific biological questions.

Despite these strengths, the present study also has limitations that define directions for future work. The biological validation was intentionally restricted to a single adipose–immune model and basic tracer-transport studies; additional applications will be needed to fully chart the operating space of the platform with respect to flow rates, shear tolerance, long-term viability, and compatibility with primary human tissues or organoids. Quantitative modeling of mass transport across the porous barriers, incorporating both diffusion and convection, would further sharpen the predictive power of the system and aid in the rational design of chip geometries for target pharmacokinetic or signaling profiles. Integration of embedded sensors (e.g., TEER electrodes, oxygen and metabolite sensors) and on-chip analytical modules could transform these devices from static culture platforms into self-reporting microphysiological systems suitable for high-content, time-resolved studies and medium-throughput screening. Finally, while U937 cells serve as an established monocyte/macrophage surrogate, they differ from primary human macrophages in transcriptional profile and inflammatory fidelity; future studies will employ primary human immune cells. Addressing these challenges will be essential to fully exploit the capabilities of the modular compartmentalized architecture and to position it as a robust, broadly adoptable platform for organotypic co-culture and multi-organ modeling.

## 5. Conclusions

This work introduces a family of versatile, modular microfluidic systems for organotypic cell co-culture, built around planar, compartmentalized architectures with tunable semi-porous side barriers. These barriers define discrete fluidic compartments while generating perfusion microchannels that sustain controlled medium flow and regulated molecular exchange between neighboring cell populations. By flexibly arranging multiple chambers in close proximity, ranging from simple two-compartment layouts to more complex interdigitated, honeycomb, and tri-compartment geometries, the platform supports the co-culture of distinct cell types in dynamically perfused microenvironments that better recapitulate the spatial organization and functional coupling of human tissues. The proof-of-concept adipose–immune model demonstrates that these devices can reproduce key features of immunometabolic crosstalk, underscoring their relevance for disease modeling. Collectively, the presented systems provide an engineering framework for constructing customizable organ- and multi-organ-on-chip models, with strong potential to deepen our understanding of organ physiology, pathophysiology, and therapeutic responses.

## Figures and Tables

**Table 1:** Inter-compartment fluid flow within the linear organization structure.

Design	Fluidic Route	Donor Compartment	Acceptor Compartment
T1	C1 → C2 → C3 → C4 → C5	C1	C2, C3, C4, C5
T2	Ci → Co	Ci	Co
T3	Ci → Co	Ci	Co
T4	Ci → C1 & C2	Ci	C1 and C2
T5	Ci → C1 & C2	Ci	C1 and C2

## Data Availability

The original contributions presented in this study are included in the article. Further inquiries can be directed to the corresponding author.
